# (2*E*)-1-[2-Hydr­oxy-4-(2-methyl­prop­oxy)phen­yl]-3-(4-methyl­phen­yl)prop-2-en-1-one

**DOI:** 10.1107/S1600536810010809

**Published:** 2010-03-27

**Authors:** Jeshal G. Maheta, Vijay M. Barot, Mukesh M. Jotani, Edward R. T. Tiekink

**Affiliations:** a Department of Chemistry, Smt. S. M. Panchal Science College, Talod, Gujarat 383 215, India; b Department of Physics, Bhavan’s Sheth R. A. College of Science, Ahmedabad, Gujarat 380 001, India; c Department of Chemistry, University of Malaya, 50603 Kuala Lumpur, Malaysia

## Abstract

The benzene rings in the title compound, C_20_H_22_O_3_, form a dihedral angle of 10.39 (8)°. Overall, the mol­ecule is approximately planar with the exception of one of the terminal methyl groups; excluding this group, the r.m.s. deviation for the remaining 22 non-H atoms is 0.0968 Å. The conformation about the C=C bond is *E*, and an intra­molecular O—H⋯O hydrogen bond leads to the formation of an *S*(6) motif. In the crystal, linear supra­molecular chains are formed along the *a* axis *via* C—H⋯O contacts, and these are connected into double chains *via* C—H⋯π inter­actions.

## Related literature

For the use of α,β-unsaturated ketones in organic synthesis, see: Marzinzik & Felder (1998 ▶); Srikanth *et al.* (2005 ▶); Nehad *et al.* (2007 ▶); Gaede & Mcdermott (1993 ▶); Shibata *et al.* (1993 ▶); Xu *et al.* (2001 ▶). For the biological activity of α,β-unsaturated ketones, see: Prasad *et al.* (2008 ▶); Zhao *et al.* (2007 ▶). Lambert *et al.* (2009 ▶); Jung *et al.* (2008 ▶); Reichwald *et al.* (2008 ▶); Boumendjel *et al.* (2008 ▶); Domínguez *et al.* (2005 ▶); Yun *et al.* (2006 ▶). For semi-empirical quantum chemical calculations, see: Stewart (2009 ▶).
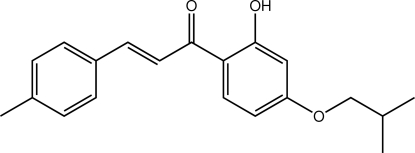


## Experimental

### 

#### Crystal data

C_20_H_22_O_3_
*M* *_r_* = 310.38
Triclinic, 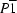

*a* = 6.7795 (8) Å
*b* = 9.8830 (12) Å
*c* = 13.9064 (17) Å
α = 74.740 (2)°
β = 78.857 (2)°
γ = 74.103 (2)°
*V* = 857.12 (18) Å^3^
*Z* = 2
Mo *K*α radiation
μ = 0.08 mm^−1^
*T* = 293 K
0.30 × 0.20 × 0.20 mm

#### Data collection

Bruker SMART APEX CCD diffractometer
Absorption correction: multi-scan (*SADABS*; Sheldrick, 1996 ▶) *T*_min_ = 0.932, *T*_max_ = 0.991
9290 measured reflections
3530 independent reflections
2452 reflections with *I* > 2σ(*I*)
*R*_int_ = 0.019

#### Refinement

*R*[*F* ^2^ > 2σ(*F* ^2^)] = 0.049
*wR*(*F* ^2^) = 0.142
*S* = 1.03
3530 reflections
212 parameters
H-atom parameters constrained
Δρ_max_ = 0.16 e Å^−3^
Δρ_min_ = −0.21 e Å^−3^

### 

Data collection: *APEX2* (Bruker, 2004 ▶); cell refinement: *APEX2* and *SAINT* (Bruker, 2004 ▶); data reduction: *SAINT* and *XPREP* (Bruker, 2004 ▶); program(s) used to solve structure: *SHELXS97* (Sheldrick, 2008 ▶); program(s) used to refine structure: *SHELXL97* (Sheldrick, 2008 ▶); molecular graphics: *ORTEP-3* (Farrugia, 1997 ▶) and *DIAMOND* (Brandenburg, 2006 ▶); software used to prepare material for publication: *publCIF* (Westrip, 2010 ▶).

## Supplementary Material

Crystal structure: contains datablocks global, I. DOI: 10.1107/S1600536810010809/pk2236sup1.cif

Structure factors: contains datablocks I. DOI: 10.1107/S1600536810010809/pk2236Isup2.hkl

Additional supplementary materials:  crystallographic information; 3D view; checkCIF report

## Figures and Tables

**Table 1:** Hydrogen-bond geometry (Å, °) *Cg* is the centroid of the C2–C7 ring.

*D*—H⋯*A*	*D*—H	H⋯*A*	*D*⋯*A*	*D*—H⋯*A*
O2—H2*o*⋯O1	0.82	1.77	2.499 (2)	148
C12—H12⋯O2^i^	0.93	2.55	3.268 (2)	135
C17—H17b⋯*Cg*^ii^	0.97	2.82	3.705 (2)	153

Symmetry codes: (i) 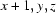; (ii) 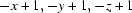.

**Fig. 1.**
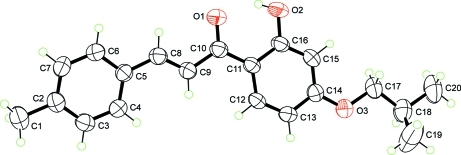
The molecular structure of (I) showing the atom-labelling scheme and displacement ellipsoids at the 50% probability level.

**Fig. 2.**
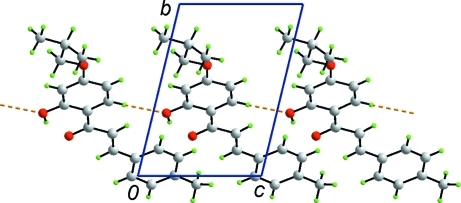
A supramolecular chain aligned along the a axis in (I), mediated by C–H···O interactions (orange dashed lines). Colour code: O, red; C, grey; and H, green.

**Fig. 3.**
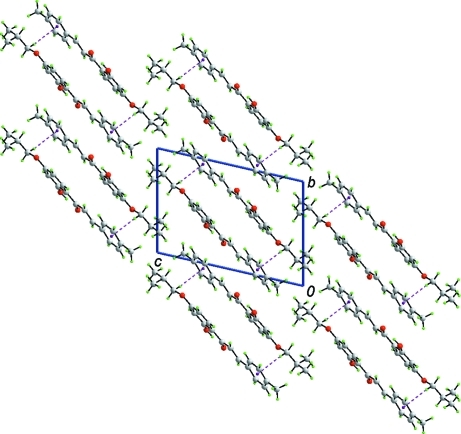
A view in projection along the a axis of the unit cell contents in (I), showing C–H···π interactions (purple dashed lines) between the supramolecular chains illustrated in Fig. 2. Colour code: O, red; C, grey; and H, green.

## supplementary crystallographic information

### Comment

α,β-Unsaturated ketones (chalcones) are useful key intermediates in organic synthesis (Marzinzik & Felder, 1998; Srikanth *et al.*, 2005). For example, they attract interest owing to their utility as starting materials in the synthesis of the five- (Nehad *et al.*, 2007 & Gaede & Mcdermott, 1993), six- (Shibata *et al.*, 1993), and seven-membered (Xu *et al.*, 2001) heterocycles. Several analogues have been demonstrated to be active against both gram-positive and gram-negative bacterial strains, and also against fungal strains (Prasad *et al.*, 2008; Zhao *et al.*, 2007). Moreover, chalcones possess a wide spectrum of biological activities such as anti-oxidant, neuroprotective, anti-leishmanial, anti-mitotic, anti-malarial, anti-cancer, etc. (Lambert *et al.*, 2009; Jung *et al.*, 2008; Reichwald *et al.*, 2008; Boumendjel *et al.*, 2008; Domínguez *et al.*, 200; Yun *et al.*, 2006). In view of the importance of these compounds, the crystal structure of title compound, (I), was determined.

With the exception of the methyl-C19 atom, the molecular structure, Fig. 1, is essentially planar. Thus, the r.m.s. deviations of the 22 non-H atoms is 0.0968 Å [maximum deviation = 0.2173 (15) Å for the C6 atom] with the C19 atom lying 1.313 (4) Å out of this plane. The near planarity is manifested in the torsion angles with the maximum deviations from linearity (excluding that involving the C19 atom; O3–C17–C18–C19 = -58.7 (2) °) found in the C8–C9–C10–O1 and C8–C9–C10–C11 torsion angles of -6.2 (2) and 173.89 (14) °, respectively. The dihedral angle formed between the benzene rings is 10.39 (8) °. The conformation about the C8═C9 bond [1.325 (2) Å] is *E*. The presence of an intramolecular *O*–*H*···O_carbonyl_ hydrogen bond is noted, Table 1, which closes an S(6) motif.

In the crystal packing, both C–H···O and C–H···π interactions are observed. Linear supramolecular chains aligned along the *a* axis are mediated by C–H···O contacts, Fig. 2 and Table 1. Centrosymmetrically related pairs of these chains are connected into a double chain via C–H···π contacts formed between the methylene-C17—H and the ring centroid of the tolyl ring, Fig. 3 and Table 1.

Semi-empirical Quantum Chemical Calculations were performed on (I) using the MOPAC2009 program (Stewart, 2009) to optimize the structure with the Parameterization Model 6 (PM6) approximation together with the restricted Hartree-Fock closed-shell wavefunction. Minimizations were terminated at an r.m.s. gradient of less than 0.01 kJ mol-1 Å^-1^. The geometry optimised structure displays a significant difference in the relative orientation of the tolyl ring compared with the experimental structure. This is quantified by the value of the C6—C5—C8—C9 torsion angle of 150.7 compared with the experimental value of 179.72 (15) °. This change is related to the participation of this ring in the C–H···π contact as discussed above.

### Experimental

A mixture of 2-hydroxy-4-isobutoxy acetophenone (0.01 mol) and 4-methyl benzaldehyde (0.01 mol) in ethanol (40 ml) were placed in a 250 ml round bottom flask and the resulting solution stirred at room temperature. After the solution became clear, a solution of potassium hydroxide (40%, 40 ml) was added slowly with constant stirring followed by stirring at room temperature for a further 20 h. After the completion of reaction, as indicated by TLC, the contents were poured onto crushed ice and acidified with dilute HCl (10%). The solid separated and was washed with water, filtered, and the crude product was crystallized from methanol to obtain (I) in 90 % yield; m.pt. 417 K. The yellow needles were obtained by the slow evaporation of a methanol solution of (I).

### Refinement

The H atoms were placed geometrically (O–H = 0.83 Å and C–H = 0.93–0.98 Å) and refined as riding with *U_iso_*(H) = 1.2–1.5*U_eq_*(parent atom).

### Crystal data

**Table:**

C_20_H_22_O_3_	*Z* = 2
*M**_r_* = 310.38	*F*(000) = 332
Triclinic, *P*1	*D*_x_ = 1.203 Mg m^−^^3^
Hall symbol: -P 1	Mo *K*α radiation, λ = 0.71073 Å
*a* = 6.7795 (8) Å	Cell parameters from 3222 reflections
*b* = 9.8830 (12) Å	θ = 2.8–24.2°
*c* = 13.9064 (17) Å	µ = 0.08 mm^−^^1^
α = 74.740 (2)°	*T* = 293 K
β = 78.857 (2)°	Block, colourless
γ = 74.103 (2)°	0.30 × 0.20 × 0.20 mm
*V* = 857.12 (18) Å^3^	

### Data collection

**Table:**

Bruker SMART APEX CCD diffractometer	3530 independent reflections
Radiation source: fine-focus sealed tube	2452 reflections with *I* > 2σ(*I*)
graphite	*R*_int_ = 0.019
ω and φ scans	θ_max_ = 26.5°, θ_min_ = 2.2°
Absorption correction: multi-scan (*SADABS*; Sheldrick, 1996)	*h* = −8→8
*T*_min_ = 0.932, *T*_max_ = 0.991	*k* = −12→12
9290 measured reflections	*l* = −17→17

### Refinement

**Table:**

Refinement on *F*^2^	Primary atom site location: structure-invariant direct methods
Least-squares matrix: full	Secondary atom site location: difference Fourier map
*R*[*F*^2^ > 2σ(*F*^2^)] = 0.049	Hydrogen site location: inferred from neighbouring sites
*wR*(*F*^2^) = 0.142	H-atom parameters constrained
*S* = 1.03	*w* = 1/[σ^2^(*F*_o_^2^) + (0.067*P*)^2^ + 0.1242*P*] where *P* = (*F*_o_^2^ + 2*F*_c_^2^)/3
3530 reflections	(Δ/σ)_max_ = 0.001
212 parameters	Δρ_max_ = 0.16 e Å^−^^3^
0 restraints	Δρ_min_ = −0.21 e Å^−^^3^

### Special details

**Table:**

Geometry. All s.u.'s (except the s.u. in the dihedral angle between two l.s. planes) are estimated using the full covariance matrix. The cell s.u.'s are taken into account individually in the estimation of s.u.'s in distances, angles and torsion angles; correlations between s.u.'s in cell parameters are only used when they are defined by crystal symmetry. An approximate (isotropic) treatment of cell s.u.'s is used for estimating s.u.'s involving l.s. planes.
Refinement. Refinement of *F*^2^ against ALL reflections. The weighted *R*-factor wR and goodness of fit *S* are based on *F*^2^, conventional *R*-factors *R* are based on *F*, with *F* set to zero for negative *F*^2^. The threshold expression of *F*^2^ > 2σ(*F*^2^) is used only for calculating *R*-factors(gt) etc. and is not relevant to the choice of reflections for refinement. *R*-factors based on *F*^2^ are statistically about twice as large as those based on *F*, and *R*- factors based on ALL data will be even larger.

### Fractional atomic coordinates and isotropic or equivalent isotropic displacement parameters (Å^2^)

**Table:**

	*x*	*y*	*z*	*U*_iso_*/*U*_eq_	
O1	0.36395 (17)	0.23227 (14)	0.54491 (10)	0.0693 (4)	
O2	0.10785 (17)	0.36469 (16)	0.66378 (11)	0.0787 (4)	
H2o	0.1506	0.3135	0.6228	0.118*	
O3	0.34346 (17)	0.64303 (13)	0.82850 (9)	0.0630 (3)	
C1	1.4896 (3)	−0.0477 (2)	0.18171 (15)	0.0735 (5)	
H1A	1.4928	−0.1427	0.1753	0.110*	
H1B	1.6102	−0.0506	0.2094	0.110*	
H1C	1.4874	0.0170	0.1167	0.110*	
C2	1.2985 (2)	0.00433 (18)	0.25032 (12)	0.0541 (4)	
C3	1.2904 (2)	0.10836 (18)	0.30244 (13)	0.0571 (4)	
H3	1.4053	0.1463	0.2947	0.069*	
C4	1.1166 (2)	0.15668 (17)	0.36524 (12)	0.0531 (4)	
H4	1.1164	0.2257	0.3997	0.064*	
C5	0.9411 (2)	0.10332 (16)	0.37782 (11)	0.0460 (4)	
C6	0.9496 (2)	−0.00067 (17)	0.32580 (12)	0.0529 (4)	
H6	0.8345	−0.0381	0.3329	0.063*	
C7	1.1248 (3)	−0.04960 (18)	0.26386 (12)	0.0565 (4)	
H7	1.1264	−0.1202	0.2306	0.068*	
C8	0.7507 (2)	0.15052 (17)	0.44205 (11)	0.0500 (4)	
H8	0.6440	0.1072	0.4439	0.060*	
C9	0.7096 (2)	0.24698 (16)	0.49809 (11)	0.0487 (4)	
H9	0.8116	0.2920	0.5008	0.058*	
C10	0.5048 (2)	0.28363 (17)	0.55592 (12)	0.0489 (4)	
C11	0.4634 (2)	0.37884 (16)	0.62556 (11)	0.0443 (4)	
C12	0.6143 (2)	0.43552 (16)	0.64662 (12)	0.0482 (4)	
H12	0.7483	0.4135	0.6138	0.058*	
C13	0.5715 (2)	0.52170 (18)	0.71343 (12)	0.0527 (4)	
H13	0.6752	0.5579	0.7255	0.063*	
C14	0.3717 (2)	0.55579 (16)	0.76392 (11)	0.0486 (4)	
C15	0.2186 (2)	0.50210 (17)	0.74614 (12)	0.0519 (4)	
H15	0.0856	0.5244	0.7799	0.062*	
C16	0.2630 (2)	0.41471 (17)	0.67776 (12)	0.0500 (4)	
C17	0.1455 (3)	0.6734 (2)	0.88789 (13)	0.0624 (5)	
H17A	0.1109	0.5839	0.9265	0.075*	
H17B	0.0400	0.7242	0.8444	0.075*	
C18	0.1529 (3)	0.7644 (2)	0.95744 (15)	0.0725 (5)	
H18	0.1876	0.8539	0.9162	0.087*	
C19	0.3163 (5)	0.6912 (4)	1.0255 (2)	0.1396 (12)	
H19A	0.2890	0.6010	1.0647	0.209*	
H19B	0.3149	0.7519	1.0694	0.209*	
H19C	0.4495	0.6738	0.9858	0.209*	
C20	−0.0607 (4)	0.8042 (3)	1.01622 (17)	0.0974 (8)	
H20A	−0.1597	0.8542	0.9702	0.146*	
H20B	−0.0579	0.8656	1.0590	0.146*	
H20C	−0.0992	0.7181	1.0565	0.146*	

### Atomic displacement parameters (Å^2^)

**Table:**

	*U*^11^	*U*^22^	*U*^33^	*U*^12^	*U*^13^	*U*^23^
O1	0.0475 (7)	0.0922 (9)	0.0841 (9)	−0.0267 (6)	−0.0001 (6)	−0.0423 (7)
O2	0.0374 (6)	0.1094 (11)	0.1091 (11)	−0.0264 (7)	0.0034 (6)	−0.0578 (9)
O3	0.0544 (7)	0.0737 (8)	0.0670 (7)	−0.0152 (6)	0.0014 (6)	−0.0326 (6)
C1	0.0640 (11)	0.0825 (13)	0.0680 (12)	−0.0107 (10)	0.0067 (9)	−0.0247 (10)
C2	0.0514 (9)	0.0575 (10)	0.0483 (9)	−0.0080 (7)	−0.0034 (7)	−0.0101 (8)
C3	0.0478 (9)	0.0617 (10)	0.0646 (10)	−0.0188 (8)	−0.0031 (8)	−0.0156 (9)
C4	0.0525 (9)	0.0551 (9)	0.0568 (9)	−0.0144 (7)	−0.0067 (7)	−0.0196 (8)
C5	0.0472 (8)	0.0487 (8)	0.0423 (8)	−0.0109 (7)	−0.0079 (6)	−0.0091 (7)
C6	0.0521 (9)	0.0585 (9)	0.0540 (9)	−0.0195 (8)	−0.0066 (7)	−0.0162 (8)
C7	0.0646 (10)	0.0572 (10)	0.0521 (9)	−0.0135 (8)	−0.0055 (8)	−0.0219 (8)
C8	0.0453 (8)	0.0576 (9)	0.0493 (9)	−0.0149 (7)	−0.0076 (7)	−0.0115 (8)
C9	0.0428 (8)	0.0539 (9)	0.0498 (9)	−0.0121 (7)	−0.0051 (7)	−0.0121 (7)
C10	0.0419 (8)	0.0538 (9)	0.0511 (9)	−0.0117 (7)	−0.0081 (7)	−0.0102 (7)
C11	0.0347 (7)	0.0486 (8)	0.0473 (8)	−0.0080 (6)	−0.0061 (6)	−0.0082 (7)
C12	0.0323 (7)	0.0557 (9)	0.0553 (9)	−0.0094 (6)	−0.0020 (6)	−0.0138 (7)
C13	0.0403 (8)	0.0615 (10)	0.0616 (10)	−0.0154 (7)	−0.0066 (7)	−0.0193 (8)
C14	0.0466 (8)	0.0492 (9)	0.0471 (8)	−0.0079 (7)	−0.0043 (7)	−0.0109 (7)
C15	0.0366 (8)	0.0591 (10)	0.0563 (9)	−0.0092 (7)	0.0022 (7)	−0.0148 (8)
C16	0.0347 (8)	0.0574 (9)	0.0587 (9)	−0.0123 (7)	−0.0051 (7)	−0.0136 (8)
C17	0.0605 (10)	0.0658 (11)	0.0568 (10)	−0.0118 (9)	0.0061 (8)	−0.0198 (9)
C18	0.0815 (13)	0.0665 (12)	0.0679 (12)	−0.0112 (10)	0.0004 (10)	−0.0262 (10)
C19	0.144 (3)	0.168 (3)	0.119 (2)	0.027 (2)	−0.0586 (19)	−0.088 (2)
C20	0.1058 (18)	0.0937 (16)	0.0830 (15)	−0.0119 (14)	0.0221 (13)	−0.0400 (13)

### Geometric parameters (Å, °)

**Table:**

O1—C10	1.2474 (18)	C9—H9	0.9300
O2—C16	1.3429 (18)	C10—C11	1.460 (2)
O2—H2o	0.8200	C11—C12	1.401 (2)
O3—C14	1.3556 (19)	C11—C16	1.410 (2)
O3—C17	1.4332 (19)	C12—C13	1.360 (2)
C1—C2	1.506 (2)	C12—H12	0.9300
C1—H1A	0.9600	C13—C14	1.396 (2)
C1—H1B	0.9600	C13—H13	0.9300
C1—H1C	0.9600	C14—C15	1.374 (2)
C2—C7	1.383 (2)	C15—C16	1.386 (2)
C2—C3	1.390 (2)	C15—H15	0.9300
C3—C4	1.376 (2)	C17—C18	1.499 (3)
C3—H3	0.9300	C17—H17A	0.9700
C4—C5	1.393 (2)	C17—H17B	0.9700
C4—H4	0.9300	C18—C19	1.500 (3)
C5—C6	1.388 (2)	C18—C20	1.523 (3)
C5—C8	1.458 (2)	C18—H18	0.9800
C6—C7	1.377 (2)	C19—H19A	0.9600
C6—H6	0.9300	C19—H19B	0.9600
C7—H7	0.9300	C19—H19C	0.9600
C8—C9	1.325 (2)	C20—H20A	0.9600
C8—H8	0.9300	C20—H20B	0.9600
C9—C10	1.467 (2)	C20—H20C	0.9600
			
C16—O2—H2o	109.5	C13—C12—H12	118.9
C14—O3—C17	118.25 (13)	C11—C12—H12	118.9
C2—C1—H1A	109.5	C12—C13—C14	119.92 (14)
C2—C1—H1B	109.5	C12—C13—H13	120.0
H1A—C1—H1B	109.5	C14—C13—H13	120.0
C2—C1—H1C	109.5	O3—C14—C15	124.34 (14)
H1A—C1—H1C	109.5	O3—C14—C13	115.70 (14)
H1B—C1—H1C	109.5	C15—C14—C13	119.96 (14)
C7—C2—C3	117.53 (15)	C14—C15—C16	119.85 (14)
C7—C2—C1	121.29 (16)	C14—C15—H15	120.1
C3—C2—C1	121.18 (16)	C16—C15—H15	120.1
C4—C3—C2	121.64 (15)	O2—C16—C15	117.28 (13)
C4—C3—H3	119.2	O2—C16—C11	121.36 (14)
C2—C3—H3	119.2	C15—C16—C11	121.35 (14)
C3—C4—C5	120.67 (15)	O3—C17—C18	109.14 (15)
C3—C4—H4	119.7	O3—C17—H17A	109.9
C5—C4—H4	119.7	C18—C17—H17A	109.9
C6—C5—C4	117.58 (14)	O3—C17—H17B	109.9
C6—C5—C8	118.64 (14)	C18—C17—H17B	109.9
C4—C5—C8	123.78 (14)	H17A—C17—H17B	108.3
C7—C6—C5	121.43 (15)	C17—C18—C19	111.91 (17)
C7—C6—H6	119.3	C17—C18—C20	109.22 (18)
C5—C6—H6	119.3	C19—C18—C20	112.0 (2)
C6—C7—C2	121.14 (15)	C17—C18—H18	107.8
C6—C7—H7	119.4	C19—C18—H18	107.8
C2—C7—H7	119.4	C20—C18—H18	107.8
C9—C8—C5	128.63 (15)	C18—C19—H19A	109.5
C9—C8—H8	115.7	C18—C19—H19B	109.5
C5—C8—H8	115.7	H19A—C19—H19B	109.5
C8—C9—C10	120.73 (14)	C18—C19—H19C	109.5
C8—C9—H9	119.6	H19A—C19—H19C	109.5
C10—C9—H9	119.6	H19B—C19—H19C	109.5
O1—C10—C11	119.71 (14)	C18—C20—H20A	109.5
O1—C10—C9	119.01 (14)	C18—C20—H20B	109.5
C11—C10—C9	121.27 (13)	H20A—C20—H20B	109.5
C12—C11—C16	116.67 (14)	C18—C20—H20C	109.5
C12—C11—C10	123.71 (13)	H20A—C20—H20C	109.5
C16—C11—C10	119.59 (13)	H20B—C20—H20C	109.5
C13—C12—C11	122.25 (14)		
			
C7—C2—C3—C4	−0.1 (3)	C16—C11—C12—C13	−0.4 (2)
C1—C2—C3—C4	179.96 (15)	C10—C11—C12—C13	−178.68 (14)
C2—C3—C4—C5	−0.7 (3)	C11—C12—C13—C14	0.3 (2)
C3—C4—C5—C6	0.7 (2)	C17—O3—C14—C15	4.8 (2)
C3—C4—C5—C8	−179.25 (14)	C17—O3—C14—C13	−175.59 (14)
C4—C5—C6—C7	0.0 (2)	C12—C13—C14—O3	−179.59 (13)
C8—C5—C6—C7	179.92 (14)	C12—C13—C14—C15	0.1 (2)
C5—C6—C7—C2	−0.7 (2)	O3—C14—C15—C16	179.37 (14)
C3—C2—C7—C6	0.7 (2)	C13—C14—C15—C16	−0.3 (2)
C1—C2—C7—C6	−179.29 (15)	C14—C15—C16—O2	179.65 (15)
C6—C5—C8—C9	179.72 (15)	C14—C15—C16—C11	0.1 (2)
C4—C5—C8—C9	−0.3 (3)	C12—C11—C16—O2	−179.30 (15)
C5—C8—C9—C10	178.15 (14)	C10—C11—C16—O2	−0.9 (2)
C8—C9—C10—O1	−6.2 (2)	C12—C11—C16—C15	0.2 (2)
C8—C9—C10—C11	173.89 (14)	C10—C11—C16—C15	178.55 (14)
O1—C10—C11—C12	176.62 (15)	C14—O3—C17—C18	176.86 (14)
C9—C10—C11—C12	−3.5 (2)	O3—C17—C18—C19	−58.7 (2)
O1—C10—C11—C16	−1.6 (2)	O3—C17—C18—C20	176.68 (15)
C9—C10—C11—C16	178.27 (13)		

### Hydrogen-bond geometry (Å, °)

**Table:**

Cg is the centroid of the C2–C7 ring.

**Table:**

*D*—H···*A*	*D*—H	H···*A*	*D*···*A*	*D*—H···*A*
O2—H2o···O1	0.82	1.77	2.499 (2)	148
C12—H12···O2^i^	0.93	2.55	3.268 (2)	135
C17—H17b···Cg^ii^	0.97	2.82	3.705 (2)	153

Symmetry codes:  (i) *x*+1, *y*, *z*; (ii) −*x*+1, −*y*+1, −*z*+1.

## References

[bb1] Boumendjel, A., Boccard, J., Carrupt, P. N., Nicolle, E., Blanc, M., Geze, A., Choisnard, L., Wouessidjewe, D., Matera, E.-L. & Dumontet, C. (2008). *J. Med. Chem* **51**, 2307–2310. 10.1021/jm0708331 18293907

[bb2] Brandenburg, K. (2006). *DIAMOND* Crystal Impact GbR, Bonn, Germany.

[bb3] Bruker (2004). *APEX2*, *SAINT* and *XPREP* Bruker AXS Inc., Madison, Wisconsin, USA.

[bb4] Domínguez, J. N., León, C., Rodrigues, J., Gamboa de Domínguez, N., Gut, J. & Rosenthal, P. J. (2005). *J. Med. Chem* **48**, 3654–3658. 10.1021/jm058208o 15887974

[bb5] Farrugia, L. J. (1997). *J. Appl. Cryst.* **30**, 565.

[bb6] Gaede, B. J. & Mcdermott, L. L. (1993). *J. Heterocycl. Chem* **30**, 49–54.

[bb7] Jung, J.-C., Jang, S., Lee, Y., Min, D., Lim, E., Jung, H., Oh, M., Oh, S. & Jung, M. (2008). *J. Med. Chem* **51**, 4054–4058. 10.1021/jm800221g 18517185

[bb8] Lambert, D. M., Aichaoui, H., Guenadil, F., Kapanda, C. N., Poupaert, J. H. & McCurdy, C. R. (2009). *Med. Chem. Res* **18**, 467–476.

[bb9] Marzinzik, A. L. & Felder, E. R. (1998). *J. Org. Chem* **63**, 723–727. 10.1021/jo971620u 11672066

[bb10] Nehad, A., El-Latif, A., Amr, A. E.-G. E. & Ibrahiem, A. A. (2007). *Monatsh. Chem* **138**, 559–567.

[bb11] Prasad, Y. R., Kumar, P. R., Smiles, D. J. & Babu, P. A. (2008). *ARKIVOC*, **11**, 266–276.

[bb12] Reichwald, C., Shimony, O., Dunkel, U., Sacerdoti-Sierra, N., Jaffe, C. L. & Kunick, C. (2008). *J. Med. Chem* **51**, 659–665. 10.1021/jm7012166 18186603

[bb13] Sheldrick, G. M. (1996). *SADABS* University of Göttingen, Germany.

[bb14] Sheldrick, G. M. (2008). *Acta Cryst.* A**64**, 112–122. 10.1107/S0108767307043930 18156677

[bb15] Shibata, K., Katsuyama, I., Izoe, H., Matsui, M. & Muramatsu, H. (1993). *J. Heterocycl. Chem* **30**, 277–281.

[bb16] Srikanth, G. S. C. & Castle, S. L. (2005). *Tetrahedron*, **61**, 10377–10441.

[bb17] Stewart, J. P. (2009). *MOPAC2009* Stewart Computational Chemistry. Available from: http://OpenMOPAC.net.

[bb18] Westrip, S. P. (2010). *publCIF* In preparation.

[bb19] Xu, J., Wang, C. & Zhang, Q. (2001). *Heteroat. Chem* **6**, 557–559.

[bb20] Yun, J.-M., Kweon, M.-H., Kwon, H., Hwang, J.-K. & Mukhtar, H. (2006). *Carcinogenesis*, **27**, 1454–1464. 10.1093/carcin/bgi348 16497706

[bb21] Zhao, P. L., Liu, C. L., Huang, W., Wang, Y. Z. & Yang, G. F. (2007). *J. Agric. Food Chem* **55**, 5697–5700. 10.1021/jf071064x 17579441

